# The *tall order* of stature estimation in burnt skeletal remains: a performance test in the Portuguese population

**DOI:** 10.1007/s00414-025-03567-2

**Published:** 2025-07-22

**Authors:** M. Beatriz Barreiro, João Fernandes, Miguel Morgado, David Gonçalves, Maria Teresa Ferreira

**Affiliations:** 1 Centre for Functional Ecology, Laboratory of Forensic Anthropology, Department of Life Sciences, University of Coimbra, Calçada Martim de Freitas, Coimbra, 3000-456 Portugal; 2 Department of Physics, Faculty of Sciences and Technology, LIBPhys-UC, University of Coimbra, Rua Larga, Coimbra, 3004-516 Portugal; 3 Archaeosciences Laboratory, Património Cultural I.P, Lisboa, Portugal; 4 Research Centre for Anthropology and Health, Department of Life Sciences, University of Coimbra, Calçada Martim de Freitas, Coimbra, 3000-456 Portugal

**Keywords:** Heat-Induced changes, Stature Estimation, Bone shrinkage, Temperature, Forensic anthropology

## Abstract

This study evaluates the performance of two stature estimation methods developed for unburnt bones – Cordeiro et al. (2009) and Mendonça (2000) – here applied to bones submitted to heat exposure. The skeletal remains of twelve individuals were experimentally burnt in an electric muffle at maximum temperatures ranging from 413ºC to 900ºC. Stature estimations were calculated before and after burning using the humerus, the femur, the first metatarsal, and the second metatarsal. Results showed that the bones burnt below 700ºC experienced minimal shrinkage (1.32 ± 0.62%), therefore resulting in post-burning interval estimates consistent with pre-burning estimates. Conversely, bones burnt at 700ºC or higher displayed accentuated shrinkage (9.57 ± 0.69%), thus affecting the performance of both methods. Heat-induced changes to the humerus and the femur frequently hindered the application of the method of Mendonça (2000), suggesting Cordeiro et al. (2009) to be more viable for burnt remains contexts. Adding a 10% shrinkage correction factor to the measurements improved the accuracy of the stature estimates, fitting the pre-burning estimates within the post-burning intervals. A 12% correction factor was also tested but it tended to overestimate the post-burning intervals. Although the method of Cordeiro et al. (2009) demonstrated a good performance when applied to burnt remains, these findings emphasize the impact of heat-induced changes on stature estimation and the necessity of specific (or adjusted) approaches for calcined bones.

## Introduction

The analysis of burnt human remains poses a major challenge for forensic anthropology due to the heat-induced changes these bones display. Particularly, fragmentation and dimensional alterations frequently affect the application of methods used for biological profile estimation [1–3]. Since dimensional changes can impact standard measurements, they can affect both metric and morphological techniques [3–7] and potentially hinder an accurate estimation of the biological profile [8, 9].

Bones exposed to heat undergo changes commonly linked to four heat-degradation stages: dehydration, decomposition, inversion, and fusion [4, 8, 10, 11]. Dimensional changes mainly take place during the inversion and fusion stages [4]. Nevertheless, bone has also been reported to expand, predominantly at lower temperatures [2, 12, 13].

Stature estimation is based solely on metric methods. Therefore, this parameter of the biological profile may be greatly affected by heat exposure, with heat-induced bone dimensional changes (and fragmentation) greatly skewing the application of traditional methods.

Despite heat-induced shrinkage being the topic of interest of a large number of studies (e.g., [1, 12, 14–17]) stature estimation in burnt bones is an underdeveloped subject. To our knowledge, the papers from Gralla [18], Strzałko et al. [19], and Rösing [20] have, for years, been the few existing attempts to develop stature estimation methods. However, they lack sufficient validation [21].

Recently, Wolska et al. [21] evaluated the accuracy of stature estimation in experimentally burnt skeletons using three different methods: the Rösing [20] method, the use of a shrinkage correction factor [19], and *chemosteometry* [22]. The Rösing [20] method involves correlating the head diameters of calcined bones, specifically the humerus, radius, and femur, to the stature of the corresponding individual. This method utilizes a nomogram that combines a 12% heat-induced shrinkage correction factor and regression equations to reconstruct bone length from head diameter, ultimately estimating living stature [20, 21]. The 10% correction factor involves adding that value to the measurements of calcined bones to account for heat-induced shrinkage. After applying this correction, sex-specific regression formulas are used to predict bone length, followed by additional regression formulas to estimate living stature from the reconstructed bone lengths [21]. The *chemosteometric* approach proposed by Gonçalves et al. [22] uses infrared spectroscopy to analyse the molecular structure of the bones, allowing for a more precise estimation of the amount of shrinkage each bone has experienced due to burning. Unlike fixed correction factors, the *chemosteometric* method provides a case-by-case assessment of shrinkage, which can lead to more accurate stature estimations from burnt skeletal remains [21].

In both forensic and biological anthropology, population specificity is a crucial aspect to consider when applying methods to estimate the biological profile, and particularly for stature estimation. Employing population-specific techniques enhances the reliability and validity of the results [3, 23]. In Portugal, the commonly used methods for stature estimation in unburnt skeletal remains are based on the femur (Mendonça [24]) and the metatarsals (Cordeiro et al. [25]), which were developed using a sample of the Portuguese population. Similar to all the other well-established stature estimation methods, these were developed using unburnt bones (more specifically, fresh bones from autopsies) and were thus designed to be used on unburnt bones as well. Therefore, applying these methods to burnt remains may yield inaccurate results since heat-induced shrinkage, warping and fragmentation might skew the results or hamper their application. Their applicability to bones with heat-induced changes has not yet been explored. Therefore, this study aimed to evaluate the performance of those two stature estimation methods in experimentally burnt skeletal remains, both alone and in combination with the 10% and 12% shrinkage correction factors.

## Materials and methods

### Sample

Twelve femora, humeri, first metatarsals, and second metatarsals (right antimeres) were experimentally burnt in an electric furnace (Barracha K-3) with a type K probe (negative: nickel-aluminium, positive: nickel-chrome) following norm IEC 60584-2. These bones belong to twelve adult individuals from the sub-collection of experimentally burnt skeletons, stemming from the 21 st Century Identified Skeletal Collection (CEI/XXI), housed at the Laboratory of Forensic Anthropology, Department of Life Sciences, University of Coimbra [26, 27]. Research done in the collection is carried out under a legal decision from the Ethics Committee of the Faculty of Medicine of the University of Coimbra (CE_026.2016).

Of these 12 skeletons, eight were from female individuals with an age-at-death ranging from 64 to 96 years (mean age = 84.63 ± 9.62 years). The remaining four skeletons were from male individuals with an age-at-death ranging from 74 to 94 years (mean age = 85.25 ± 8.20 years). The experimental burns were performed following the protocol described in Gonçalves et al. [27], which encompasses a photographic record of the bones, recording mass and several metric measurements, and bone powder sampling, before and after burning. Maximum temperatures ranged from 413 °C to 900 °C, and burn durations varied between 42 min and 310 min. The sample was divided into two groups, one group comprising the skeletons burnt at maximum temperatures < 700 °C (*n* = 7), thus corresponding roughly to non-calcined burnt bones; and the other comprising the skeletons burnt at maximum temperatures ≥ 700ºC (*n* = 5), thus corresponding roughly to calcined bones (Table 1).

**Table 1:** Sex, age-at-death and number of CEI/XXI individuals, grouped by maximum temperature. Burning temperature and duration ranges are also given per sex per temperature group

	Sex	*N*	Age-at-death	Mean	S.D	Temperature (°C)	Duration (min)
<700 °C	Female	5	64–96	84.20	11.57	450–500	157–294
	Male	2	81–92	86.50	5.50	413–450	42–210
≥700 °C	Female	3	81–92	85.33	4.78	700–800	237–300
	Male	2	74–94	84.00	10.00	700–900	194–310

### Methods

The Mendonça [24] and Cordeiro et al. [25] methods were applied to the sample before and after burning, to obtain stature estimates.

Mendonça [24] produces stature estimates based on standard measurements of the humerus and femur according to sex (Table 2). Cordeiro et al. [25] uses measurements of the first and second metatarsals, providing sex-specific and unknown sex formulas (Table 2). The unknown sex formulas were used when applying Cordeiro et al. [25], whilst sex-specific formulas were used with Mendonça [24]. Even though the sex of the skeletons used in this study is known, the decision to use the unknown sex formulas of Cordeiro et al. [25] was based on the fact that, in real situations, sex might not be known or possible to estimate (especially, if the metatarsals are the only available remains).

Dimensional changes between the pre-burnt and burnt stages of each bone were evaluated for the standard measurements detailed in Table 2. Bone shrinkage (%) was calculated according to the following formula proposed by Shipman et al. [16]:

$$\begin{aligned}&\varvec{S}\varvec{h}\varvec{r}\varvec{i}\varvec{n}\varvec{k}\varvec{a}\varvec{g}\varvec{e}\:\left(\varvec{\%}\right)=\\&\frac{\left(Pre-burning\:dimension\right)-(Post-burning\:dimension)\:}{(Pre-burning\:dimension)}\:\times\:100\end{aligned}$$

**Fig. 1:**
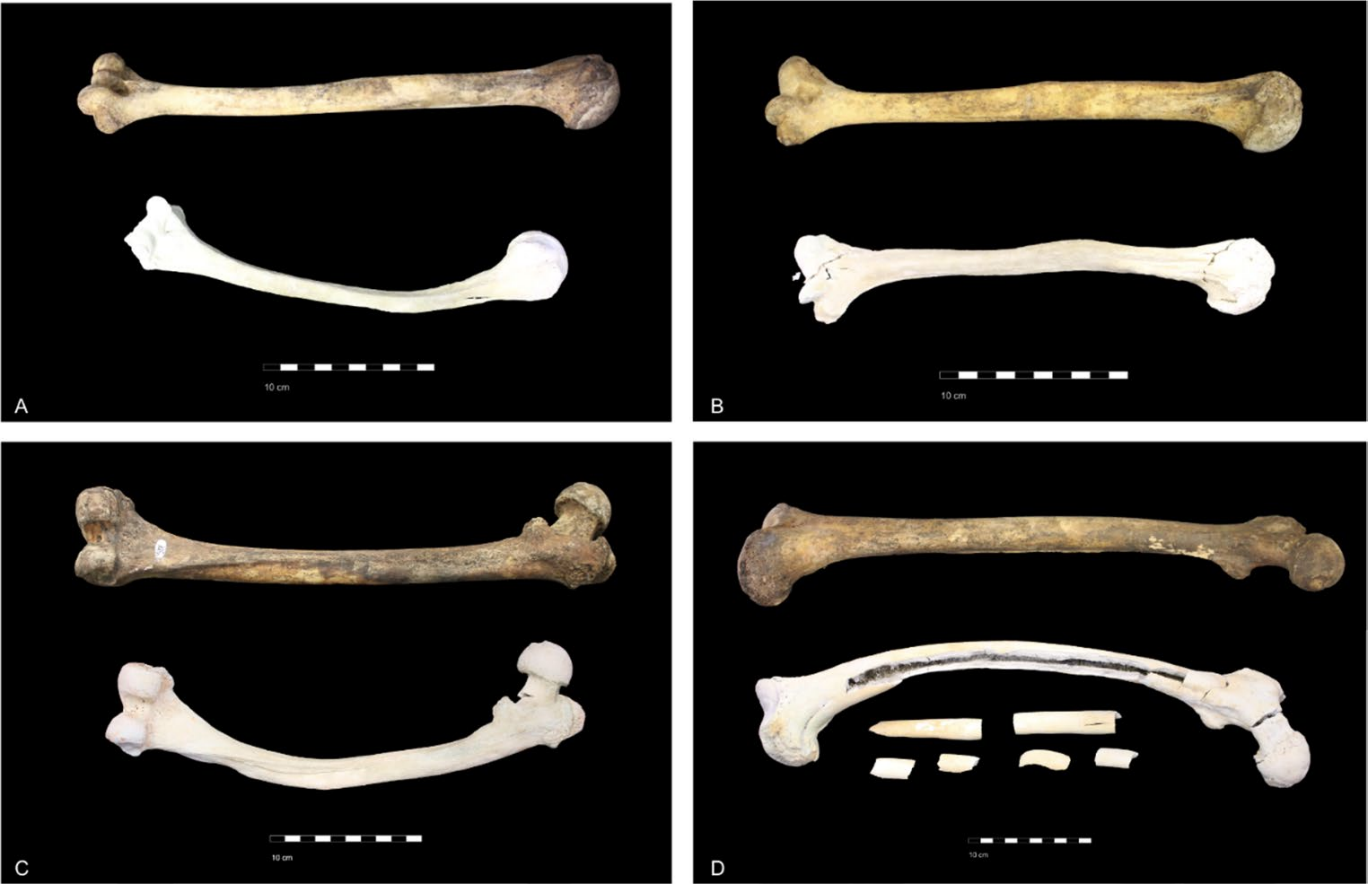
Humeri and femora exemplifying the most accentuated heat-induced changes in the sample. The humerus and femora depicted in A, C, and D exhibit extreme warping. The humerus depicted in B displays the most accentuated shrinkage observed in the sample. **A** – Humerus burnt at 700ºC for 300 min; **B** – Humerus burnt at 800ºC for 255 min; **C** – Femur burnt at 700ºC for 237 min; and **D** – Femur burnt at 700ºC for 194 min. The same bone is presented in each image before (top) and after (bottom) burning for comparison of the original dimensions

By using this formula, positive results referred to shrinkage while negative results referred to expansion. It should be noted that the term “bone shrinkage” is here used loosely to describe heat-induced dimensional changes caused by both true shrinkage and warping. Although the latter was negligible in the case of metatarsal bones, it was sometimes quite drastic in the case of femora and humeri (See Fig. 1 in the results section). Notwithstanding this having a clear impact on their measurement, we decided to use them as examples of extreme cases of dimensional change to obtain a broader picture of stature estimation reliability.

The impact of thermally induced changes on these methods was assessed by analysing if the pre-burning stature estimate was contained within the post-burning stature interval calculated from the standard errors of each linear regression equation. This approach was chosen because it mimics a forensic case more closely. The pre-burning estimate was used here as a proxy for the recorded stature of the living individual to be compared against the estimation interval obtained during the post-mortem examination.

Additionally, shrinkage correction factors of 10% and 12% were applied to the post-burning measurements of the calcined bones to estimate the pre-burn metric dimensions. Post-burning stature estimates were then recalculated using the adjusted measurements and again compared to the pre-burning estimation interval.

The measurements described in Table 2 were recorded before and after burning by three observers with different levels of experience and academic degrees. The coefficient of variation (CV) was used to assess inter-observer variability.

**Table 2:** Stature estimation formulas provided by mendonça [24] and Cordeiro et al. [25]

Method	Measurement (mm)	Formulas		
		Males	Females	Unknown sex
Mendonça (2000) [24]	Full Length of the Humerus (FLH)	STAT = 59.41+0.3269FLH ±8.44	STAT = 64.26+0.3065 FLH ±7.70	
	Physiological Length of the Femur (PhLF)	STAT = 47.18+0.2663PhLF ±6.90	STAT = 55.63+0.2428PhLF ±5.92	
	Perpendicular Length of the Femur (PLF)	STAT = 46.89+0.2657PLF ±6.96	STAT = 57.86+0.2359PLF ±5.96	
Cordeiro et al. (2009) [25]	Physiological Length of the 1st Metatarsal (F1)	S=963.949+11.678F1 ±57.0	S=919.146+12.00F1 ±43.5	S=887.530+12.826F1 ±55.2
	Physiological Length of the 2nd Metatarsal (F2)	S=834.630+11.563F2 ±47.2	S=957.350+9.488F2 ±47.0	S=798.894+11.990F2 ±47.6
	Maximum Length of the 1st Metatarsal (M1)	S=865.335+12.317M1 ±55.3	S=871.260+11.970M1 ±46.9	S=816.157+13.007M1 ±53.7
	Maximum Length of the 2nd Metatarsal (M2)	S=817.849+11.374M2 ±47.1	S=961.592+9.117M2 ±47.6	S=790.041+11.689M2 ±47.5

*STAT* = stature estimation (cm); *S* = stature estimation (mm)

## Results

The CV for both pre- and post-burning measurements was generally low, indicating good consistency between observers (Table 3). This high level of precision demonstrates that the measurement process was reliable and consistent, regardless of the observer or the condition of the bone (burnt or unburnt).

**Table 3:** Mean coefficient of variation (%), and respective standard deviation (S.D.), minimum and maximum values obtained before and after burning for the measurements recorded

	Pre-Burning				Post-Burning			
	mean CV	S.D.	min	max	mean CV	S.D.	min	max
*FLH*	0.24%	0.002	0.00%	0.55%	0.18%	0.001	0.00%	0.38%
*PLF*	0.17%	0.001	0.00%	0.42%	0.13%	0.001	0.00%	0.26%
*PhLF*	0.19%	0.001	0.00%	0.40%	0.19%	0.002	0.00%	0.51%
*M1*	0.95%	0.006	0.37%	2.16%	1.19%	0.007	0.41%	2.17%
*F1*	0.94%	0.013	0.00%	4.44%	0.80%	0.005	0.00%	1.22%
*M2*	0.56%	0.004	0.03%	1.37%	0.64%	0.005	0.18%	1.56%
*F2*	0.71%	0.006	0.00%	1.94%	0.72%	0.005	0.00%	1.54%

*FLH* - full length of the Humerus; *PLF -* perpendicular length of the Femur; *PhLF -* physiological length of the Femur; *F*1- physiological length of the 1st metatarsal; *F*2 - physiological length of the 2nd metatarsal; *M*1 - maximum length of the 1st metatarsal; *M*2 - maximum length of the 2nd metatarsal

An evaluation of the measurements used for stature estimation with the Mendonça [24] and Cordeiro et al. [25] methods revealed that dimensional changes were more pronounced at maximum temperatures of 700 °C and above, displaying a mean shrinkage of 9.57% (S.D.= 0.69). Meanwhile, minimal shrinkage was observed for bones burnt at maximum temperatures below 700 °C (mean = 1.32%; S.D.= 0.62) (Table 4). Figure 1 depicts some examples of humeri and femora with accentuated heat-induced dimensional changes, which clearly affected measurements.

**Table 4:** Bone metric changes in the < 700ºC and the ≥ 700ºC temperature groups. Mean shrinkage (%), standard deviation (S.D.), and minimum and maximum values are provided for each measurement used for stature estimation. Mean values are also provided for each temperature group. N represents the number of bones analysed

		N	mean (%)	S.D.	min (%)	max (%)
<700°C	FLH	7	0.74	0.29	0.23	1.14
	PhLF	5	0.59	0.28	0.24	0.88
	PLF	5	0.73	0.19	0.44	0.96
	F1	7	1.60	0.86	-0.01	2.61
	F2	7	1.18	0.72	0.45	2.33
	M1	7	2.47	1.39	-0.10	4.40
	M2	6	1.61	1.15	0.34	2.97
	Temperature Group	44	1.32	0.62	0.59	2.47
≥700°C	FLH	4	10.02	7.07	2.67	16.09
	PhLF	2	9.98	3.15	7.75	12.20
	PLF	3	11.10	3.28	8.84	14.86
	F1	5	9.59	3.66	6.78	15.69
	F2	5	9.18	4.05	3.79	13.74
	M1	4	9.41	2.95	5.05	11.30
	M2	5	8.65	3.74	3.96	13.21
	Temperature Group	28	9.57	0.69	8.65	11.10

*FLH -* full length of the Humerus; *PLF -* perpendicular length of the Femur; *PhLF* - Physiological length of the Femur; *F*1 - physiological length of the 1st metatarsal; *F*2 - physiological length of the 2nd metatarsal; *M*1 - maximum length of the 1st metatarsal; *M*2 - maximum length of the 2nd metatarsal

Stature estimation using Cordeiro et al. [25] performed well in all four parameters (M1, M2, F1, and F2), for temperatures below 700ºC (Table 5). In all cases, the post-burning stature intervals contained the pre-burning estimates. However, for temperatures of 700ºC and above, most pre-burning estimates (69%) fell outside the post-burning interval. Applying the 10% correction factor considerably improved the results for the majority of the cases (90%), with most of the estimates that were originally outside the post-burning intervals fitting into the estimation intervals after this correction. The 10% correction factor was particularly impactful with the F2 measurement, since all the pre-burning estimates became included within the post-burning intervals. The 12% correction factor had a similar effect, although not as positive. Also, it resulted in more cases with estimates falling outside the intervals, although that was not the case before the correction.

**Table 5:** Agreement between pre- and post-burning stature estimation results obtained using Cordeiro et al. [25] and when applied 10% and 12% correction factors. The results are grouped by burning temperature

Measurement	Temperature Group	Pre-Burning/Post-Burning Agreement		Pre-Burning/Post-Burning Agreement FC10%		Pre-Burning/Post-Burning Agreement FC12%	
		N	Percentage	N	Percentage	N	Percentage
M1	**<700°C**	7	100%				
	**≥700°C**	4	25%	4	100%	4	75%
M2	**<700°C**	6	100%				
	**≥700°C**	5	40%	5	80%	5	60%
F1	**<700°C**	7	100%				
	**≥700°C**	5	60%	5	80%	5	80%
F2	**<700°C**	7	100%				
	**≥700°C**	5	0%	5	100%	5	80%

*M*1- maximum length of the 1st metatarsal; *M*2 - maximum length of the 2nd metatarsal; *F*1- physiological length of the 1st metatarsal; *F*2 - physiological length of the 2nd metatarsal

Stature estimation using the method of Mendonça [24] also provided a good fit of the pre-burning estimates into the post-burning intervals for all the individuals burnt below 700ºC. However, the method did not perform well when applied to individuals burnt at ≥ 700ºC, as it was often either not possible to apply or, in some other cases, the pre-burning estimate fell outside the post-burning interval (Table 6). Applying the 10% and 12% correction factors reduced the difference between estimates, fitting more estimates into the intervals, with both approaches.

**Table 6:** Agreement between pre- and post-burning stature estimation results obtained by using Mendonça [24] combined with the 10% or 12% correction factors. The results are grouped by burning temperature

Measurement	Temperature Group	Pre-Burning/Post-Burning Agreement		Pre-Burning/Post-Burning Agreement FC10%		Pre-Burning/Post-Burning Agreement FC12%	
		N	Percentage	N	Percentage	N	Percentage
FLH	**<700°C**	7	100%				
	**≥700°C**	4	50%	4	75%	4	75%
PhLF	**<700°C**	5	100%				
	**≥700°C**	2	0%	2	100%	2	100%
PLF	**<700°C**	5	100%				
	**≥700°C**	3	0%	3	100%	3	100%

*FLH -* full length of the humerus; *PhLF* - physiological length of the femur; *PLF -* perpendicular length of the femur

## Discussion

This study assessed the applicability of stature estimation methods, traditionally developed (and applied) in unburnt remains, to bones with heat-induced changes. Our findings suggest that burning temperatures of ≥ 700ºC caused considerable bone shrinkage, therefore reducing the reliability and accuracy of the stature estimation methods of Cordeiro et al. [25] and Mendonça [24].

Literature shows that bones burnt at maximum temperatures of ≥ 700ºC tend to undergo major structural changes [12, 15]. Previous research has reported variable degrees of heat-induced bone metric changes, up to 40% [1, 9, 12, 16, 22, 28]. Such metric changes have been reported to have an impact on methods used to estimate the biological profile [3, 4, 22]. Our findings support these claims, reporting a mean shrinkage of approximately 10% for bones burnt at maximum temperatures of ≥ 700ºC (up to 900ºC).

The results indicate that the methods by Cordeiro et al. [25] and Mendonça [24] performed well when applied to bones burnt below 700ºC. For this temperature group, both stature estimation methods provided post-burning stature intervals that included the pre-burning stature estimates. This suggests that the heat-induced changes, particularly metric changes, on non-calcined burnt bones were not large enough to affect the reliability of both tested methodologies. In contrast, their application to calcined bones performed poorly, yielding several pre-burning estimates that fell outside the post-burning interval. However, as expected, the calcined bones displayed significant shrinkage, fractures, and fragmentation, thus affecting the reliability of the stature estimation methods. These results agree with previous work reporting that low-intensity burning conditions have no significant impact on traditional metric methods, thus allowing their application to non-calcined burnt remains, while the opposite conclusion has been reported for high-intensity burning conditions [12, 22, 29].

The two methods are based on the same approach, using linear regression formulas to estimate stature. However, their major difference lies in the bones they use for that purpose. While Mendonça [24] uses the lengths of the humerus and the femur, Cordeiro et al. [25] explores the first and second metatarsals. As observed in the present study, the femur and humerus suffered more accentuated heat-induced fractures, warping, and fragmentation that hindered measurement recording and, consequently, the application of the method of Mendonça [24]. That was particularly impactful for the higher temperatures (≥ 700ºC) but also affected a few of the estimates at < 700ºC. Stature estimations based on the humerus and the femur were surprisingly accurate even though some presented extreme heat-induced warping. This may be explained by the large estimation intervals obtained by the method from Mendonça [24] which makes it quite conservative.

On the other hand, measurements of the first and second metatarsals were never compromised by heat-induced changes. Indeed, an increased fragility and propensity for fragmentation was observed for the burnt femora and humeri compared to the first and second metatarsals. The typical brittleness of burnt bone was more easily handled by the smaller and lighter metatarsal bones than by the humeri and the femora.

Although sex was not directly taken into account for this study, it may have some effect on heat-induced shrinkage, as small variations in bone composition, density, and size between males and females have been reported [15, 30, 31]. Sex was not a factor considered here because of the small size of the sample and its heterogeneity. Nevertheless, Almeida et al. [32] and Gonçalves et al. [22] reported some differences between sexes, with the male burnt bones displaying more average shrinkage than the female bones. However, given that fragmentation and other heat-induced changes can also compromise sex estimation, Cordeiro et al. [25] still stands out as a more suitable method for burnt remains than Mendonça [24]. By providing both sex-specific and unknown sex formulas, Cordeiro et al. [25] enhances its applicability in fire-related contexts.

In summary, the method by Cordeiro et al. [25] proved more suitable than that by Mendonça [24] for burnt remains. Its application appears to be less compromised since it uses bones less prone to fragmentation and provides sex-independent formulas. Mendonça [24] requires humeri and femora to be relatively intact and the sex of the individual to be known, therefore, its applicability seems more limited under the constraints posed by burning. It is unlikely that the femoral and humeral standard measurements used by Mendonça [24] will be intact in real-world scenarios. Nonetheless, those features were used to highlight the impact of heat-induced metric changes in stature estimation. The scenario may be different for metatarsals which, based on our results, apparently tend to preserve better. In that regard, Wolska et al. [21] evaluated stature estimation on burnt remains using the transverse head diameters of humeri, femora, and radii, providing good results using bone regions that might have a better survival chance compared to the maximum lengths of the same bones.

In the absence of methods specific to calcined remains, the most viable alternative is to apply shrinkage correction factors that account for heat-induced shrinkage. The results indicated that a 10% correction factor improved the accuracy of the estimates, minimizing the differences between pre- and post-burning estimates. On the other hand, a correction factor of 12% often overcompensated, resulting in overestimated post-burning stature intervals that excluded the pre-burning estimates. As previously mentioned, a mean shrinkage of approximately 10% was recorded for maximum temperatures of ≥ 700ºC. Therefore, the correction factor of 10% appears to be a better fit, a finding also supported by Gonçalves et al. [22] and Wolska et al. [21].

While the findings of this study offer valuable insights, several limitations should be acknowledged. A key limitation of this study lies in the small sample size, which weakens our inferences. Additionally, the only factor studied here was maximum temperature. However, other factors can influence the burning process and/or the results of post-burning analysis (e.g., sex, age, burn duration) [3, 32–34]. Another potential limitation involves the pre-burning condition of the bones used in this study, i.e. previously inhumed dry bones are difficult to outline due to lack of research on this subject. The few papers addressing the role of soft tissues on heat-induced changes mainly cover warping and fractures, while no research has been conducted on the potential impact of soft tissues on heat-induced metric changes. It is known that heat-induced warping can be more prevalent on bones burnt while they were fleshed or fresh than on bones burnt dry [35, 36]. However, in that respect, our dry humeri and femora were also affected by it, sometimes even heavily affected. Therefore, we believe that the possible confounding effect caused by warping has been at least partially accounted for in our sample. In contrast, warping was much less impactful in the case of the metatarsal bones used in this study. Based on our own experience drawn from both modern crematoria with fleshed cadavers and dry skeletons studies, heat-induced warping tends to be less severe in metatarsals than other bones often used for stature estimation (e.g., femur, humerus, tibia). This factor, added to their propensity to show better preservation after heat exposure, highlights metatarsal bones as particularly good targets for stature estimation. Moreover, the age distribution of the sample is another factor that may undermine inferences, since it is mostly composed of elderly individuals (a reflection of the age demographics of CEI/XXI and also of the Portuguese mortality curve [22, 26, 27]) and therefore impact bone properties (e.g., collagen content). Furthermore, translating our findings into forensic practice is not straightforward because the intensity at which bones have been burnt needs to be determined prior to deciding if shrinkage correction factors should be applied. Fourier-transform infrared spectroscopy is a good option to assess it [22]. *Chemosteometry* may offer a good alternative to shrinkage correction factors but has not been tested here.

## Conclusion

The application of traditional stature estimation methods to bones with heat-induced changes presented some limitations. Although they performed well in bones burnt below 700ºC, the heat-induced dimensional changes, especially shrinkage experienced by bones at higher temperatures (700 ºC – 900 ºC), considerably interfered with the results. The 10% correction factor minimized the differences between pre- and post-burning estimates in calcined bones, proving to be the best approach. In turn, the 12% correction factor also improved estimates but overestimated the (post-burning) intervals.

Cordeiro et al. [25] showed better results than Mendonça [24] when applied to burnt remains due to its use of bones less prone to accentuated heat-induced changes and the inclusion of sex-independent formulas. However, since the methods investigated here were developed for unburnt skeletal remains, their performance on thermally altered bones requires further investigation to refine their reliability and validate our results.

The topic of stature estimation in burnt skeletal remains has been seldom addressed in the literature. Beyond the work of Rösing [20] and Wolska et al. [21], little hope has been granted to anthropologists regarding the possibility of estimating the stature under those conditions. For instance, until now, no stature-specific evidence from systematic research had shown that it is reliable to obtain stature estimations based on non-calcined burnt bones, by using methods developed for unburnt bones. The present work supports the notion that such approach is feasible and it contributes to broaden the discussion regarding this topic by making available new data from experimental research.

## Data Availability

The datasets generated during and/or analyzed during the current study are available from the corresponding author upon reasonable request.
